# Identification of *Escherichia coli* and *Trueperella pyogenes* isolated from the uterus of dairy cows using routine bacteriological testing and Fourier transform infrared spectroscopy

**DOI:** 10.1186/s13028-016-0262-z

**Published:** 2016-11-28

**Authors:** María Jaureguiberry, Laura Vanina Madoz, Mauricio Javier Giuliodori, Karen Wagener, Isabella Prunner, Tom Grunert, Monika Ehling-Schulz, Marc Drillich, Rodolfo Luzbel de la Sota

**Affiliations:** 1 Cátedra y Servicio de Reproducción Animal, Facultad de Ciencias Veterinarias, Universidad de La Plata (FCV-UNLP), B1900AVW La Plata, Argentina; 2 Consejo Nacional de Investigaciones Científicas y Técnicas (CONICET), C1033AAJ Buenos Aires, Argentina; 3 Cátedra de Fisiología, FCV-UNLP, B1900AVW La Plata, Argentina; 4 Institute of Microbiology, University of Veterinary Medicine Vienna, 1210 Vienna, Austria; 5 Clinical Unit for Herd Health Management in Ruminants, University Clinic for Ruminants, University of Veterinary Medicine Vienna, 1210 Vienna, Austria

**Keywords:** Bacteriological testing, Dairy cow, Fourier transform infrared spectroscopy

## Abstract

**Background:**

Uterine disorders are common postpartum diseases in dairy cows. In practice, uterine treatment is often based on systemic or locally applied antimicrobials with no previous identification of pathogens. Accurate on-farm diagnostics are not available, and routine testing is time-consuming and cost intensive. An accurate method that could simplify the identification of uterine pathogenic bacteria and improve pathogen-specific treatments could be an important advance to practitioners. The objective of the present study was to evaluate whether a database built with uterine bacteria from European dairy cows could be used to identify bacteria from Argentinean cows by Fourier transformed infrared (FTIR) spectroscopy. Uterine samples from 64 multiparous dairy cows with different types of vaginal discharge (VD) were collected between 5 and 60 days postpartum, analyzed by routine bacteriological testing methods and then re-evaluated by FTIR spectroscopy (n = 27).

**Results:**

FTIR spectroscopy identified *Escherichia coli* in 12 out of 14 samples and *Trueperella pyogenes* in 8 out of 10 samples. The agreement between the two methods was good with a Kappa coefficient of 0.73. In addition, the likelihood for bacterial growth of common uterine pathogens such as *E. coli* and *T. pyogenes* tended to increase with VD score. The odds for a positive result to *E. coli* or *T. pyogenes* was 1.88 times higher in cows with fetid VD than in herdmates with clear normal VD.

**Conclusions:**

We conclude that the presence of *E. coli* and *T. pyogenes* in uterine samples from Argentinean dairy cows can be detected with FTIR with the use of a database built with uterine bacteria from European dairy cows. Future studies are needed to determine if FTIR can be used as an alternative to routine bacteriological testing methods.

## Background

Metritis (MET), clinical endometritis (CE) and subclinical endometritis (SE) are common uterine disorders in dairy cows associated with lower reproductive performance and higher culling rates [1–3]. Risk factors for these uterine diseases mainly are problems around parturition and subsequent negative energy balance [4–6]. The most prevalent uterine pathogens isolated from cows with MET, CE, and in some cases SE are *Escherichia coli* and *Trueperella pyogenes* [7]. Other frequently isolated pathogens are *Prevotella* species, *Fusobacterium necrophorum*, and *Fusobacterium nucleatum* [8, 9]. Recent studies revealed that the post-partum uterine bacterial community is comprised of more than 200 bacterial species [10]. However, for many of these species pathogenicity and interactions between species have yet to be described. Thus, the present study focused on the well-known *E. coli* and *T. pyogenes* pathogens.

Most practitioners perform antibiotic treatment of bovine uterine infections without sampling for bacteriological testing because it is time-consuming and costly. Such an empirical treatment strategy has been proposed as a risk for the development of bacterial resistance [11]. Santos et al. [12] found that over 50% of isolated *T. pyogenes* were resistant to amoxicillin, ampicillin, chloramphenicol, florfenicol, oxytetracycline and penicillin. In addition, 35% of isolated *E. coli* showed multidrug resistance (ampicillin–chloramphenicol–florfenicol) [11]. A recent study described a potential resistance to cephapirin, which is a commonly used antibiotic to treat cows with CE in many countries [13]. The use of cephalosporins in food producing animals is a controversial issue because they are a reserve antibiotic for human medicine. In this sense, many countries have implemented measures (directive 2003/99/EC) to restrict their use to a minimum to avoid resistances [14–16].

Routine methods of bacteriological identification are based on colony characteristics, Gram staining, morphology, hemolytic ability and biochemical profile [17]. Although these methods are useful to identify bacteria, they have some limitations. The time consumed to achieve a result, the difficulty to identify some bacteria, and the need for more accurate identification of bacteria are some of these limitations. Molecular methods to identify bacteria may overcome some of these limitations.

Fourier transform infrared (FTIR) spectroscopy, a vibrational spectroscopy method that provides information about the biochemical composition (proteins, lipids, nucleic acids, polysaccharides, etc.) of the analyzed materials [18], has been established as a powerful method for bacterial identification [19]. As microorganisms differ biochemically, the spectra provided by this method serve as a fingerprint, allowing to differentiate between genus, species, strains and serotypes [20–23]. Spectral distances are indicative of the biochemical difference between microorganisms. This information is used in databases as references for future analyses of isolates. Thus, databases containing reference spectra of different strains play a key role in bacterial identification by FTIR spectroscopy. With an appropriate reference database, FTIR spectroscopy is a simple, relatively fast and inexpensive method for the identification of bacteria. However, FTIR spectroscopy is not an on-farm test and still requires shipping of samples to a laboratory.

The aim of the present study was to assess if a database containing spectra from bacteria obtained from the uterus of Austrian and German dairy cows could be used in a pilot study to identify uterine bacteria from Argentinean dairy cows by using FTIR.

## Methods

Grazing Holstein dairy cows from two commercial dairy farms located in Brandsen (35°17′S, 58°23′W, n = 34) and Castelli (36°10′S, 57°78′W, n = 30), Province of Buenos Aires, Argentina were included in the study (n = 64). The cows were between 5 and 60 days postpartum (dpp; 19 cows were below 21 dpp and 45 cows were at 21 dpp) and did not receive any antibiotic therapy in the 14 days prior to enrollment. This was designed as a convenience sampling procedure with no further selection criteria.

### Clinical examination

Cows were first examined by manual vaginal exploration after cleaning the vulva with a dry paper towel and introducing a gloved hand through the vulva [24]. Then, mucus from the cranial portion of the vagina was withdrawn for direct examination [25]. Vaginal discharge (VD) was scored as follows: VD0 = clear mucus, VD1 = clear mucus with flecks of pus, VD2 = mucopurulent non-fetid discharge and VD3 = watery, purulent or brown-colored and fetid discharge [26]. Because cows were at a wide range of dpp, the VD score was considered as a descriptive classification, but not as indicative for MET or CE according to Williams et al. [27].

### Bacteriological samples

One bacteriological sample was taken irrespective of the cows’ VD score (0–3). These samples were obtained from the endometrial surface by the cytobrush technique as described for bacteriological sampling by Westermann et al. [28]. After cleaning the vulva, the sterile brush (Medibrush XL®, Medical Engineering Co. SA; Argentina) attached to a stainless steel instrument, covered with a disposable plastic sheath (IMV Technologies, Paris, France) and protected from contamination with a sanitary plastic sleeve (SBS Cryo®Tec, Buenos Aires, Argentina), was inserted into the uterine body. Inside the uterine lumen, the sleeve was retracted and the brush was pushed forward and exteriorized from the plastic sheath to keep contact with the uterine mucosa. After that, the brush was retracted back into the sheath, and pulled out of the genital tract. Then, the brush was placed in a tube containing Stuart’s transporting medium (Eurotubo; Deltalab SL, Barcelona, Spain) and delivered in a cooling container within 6 h of sampling to the Bacteriology Laboratory at the Faculty of Veterinary Science, National University of La Plata, La Plata, Argentina. Samples were cultured for aerobic bacteria by routine bacteriological testing methods. Briefly, each brush was streaked on plates containing sheep blood agar (Laboratorios Britania, Buenos Aires, Argentina) and incubated in aerobic culture medium at 37 °C for 48 h. Bacteria were identified based on their colony characteristics, Gram staining, morphology, hemolytic ability and biochemical profile according to Winn and Koneman [17]. The bacterial colonies (n = 27) were stored at −80 °C until shipped to the Institute of Microbiology, University of Veterinary Medicine Vienna, Austria for FTIR spectroscopy analysis.

### FTIR spectroscopy

At the Institute of Microbiology, the samples were re-suspended and cultured on blood agar plates at 37 °C for 48–72 h. A subsample from each colony was cultured at 37 °C for 48 h. Identification of bacterial isolates by FTIR spectroscopy was performed as previously described [10, 29]. In brief, the isolates were cultivated as cell lawns on tryptic soy agar (Oxoid, TSA, Hampshire, UK) at 30 °C for 24 h. One loop (1 mm diameter) of cell material was suspended in 100 µL sterile deionized water by vortexing. An aliquot of 30 µL of the suspension was placed onto a ZnSe plate. After drying at 40 °C for 40 min in an oven, the FTIR measurement was carried out using a HTS-XT microplate adapter coupled to a Tensor 27 FTIR spectrometer (Bruker Optics GmbH, Ettlingen, Germany) in the transmission mode covering the spectral range from 4000–500 cm^−1^. Spectral data analysis was performed using the OPUS software (version 5.5; Bruker Optics GmbH).

The FTIR spectral library used to identify the uterine bacteria was comprised in total over 700 bacterial species [30] that included more than 200 records of bacteria isolated and identified in detail from recent samples obtained from Austrian and German cows with uterine infections [7, 10, 31].

### Statistical analyses

The degree of agreement between traditional bacteriological identification and FTIR was evaluated with Kappa’s coefficient. The likelihood for bacterial identification depending on the score of VD was assessed by logistic regression [32].

## Results

Fourteen percent of cows (9/64) were excluded from the analysis because of VD sample contamination. Therefore, data from 55 cows analyzed by routine bacteriological testing in Argentina were included. Thirty-three percent of cows had VD0, 22% had VD1, 18% had VD2, and 27% of cows had VD3. *E. coli* (n = 5) and *T. pyogenes* (n = 7) were isolated from 12 out of 34 cows in one farm, while *E. coli* (n = 9), *T. pyogenes* (n = 5)] and coagulase-negative staphylococci (CNS) (n = 1) were isolated from 15 out of 30 cows in the other farm. The bacteria were always present in monoculture.

Positive samples for *E. coli, T. pyogenes* and CNS (n = 27) were sent to the Institute of Microbiology, Vienna, Austria, where bacterial growth was achieved in 25 out of these 27 samples. Two samples without bacterial growth had been positive for *T. pyogenes* when cultured in Argentina. Thus, finally 25 samples were tested by FTIR: 14 were positive for *E. coli*, 10 for *T. pyogenes* and 1 sample for CNS.

FTIR spectroscopy identified 12 out of 14 *E. coli* positive samples as *E. coli*. The remaining 2 samples were identified as *Enterococcus faecium* and *Citrobacter freundii*. For *T. pyogenes* 8 out of the 10 positive samples were identified correctly and the remaining 2 samples were detected as *Streptococcus dysgalactiae.* Finally, the FTIR spectroscopy identified the single CNS positive sample correctly. The agreement between routine bacteriological testing methods and the FTIR spectroscopy had a Kappa’s coefficient of 0.73 (95% CI = 0.52–0.95, P < 0.001).

Based on results from routine testing in Argentina, there was a tendency to an increased likelihood of bacterial growth (*E. coli* and *T. pyogenes*) with increasing VD score (P = 0.06), given that the percentage of positive samples were 39% (7/18) in VD0, 25% (3/12) in VD1, 60% (6/10) in VD2, and 73% (11/15) in VD3. The likelihood for *E. coli* or *T. pyogenes* positive results was 1.88 times higher in a cow with VD3 than in a cow with VD0 (Table 1).

**Table 1:** Effect of vaginal discharge score on the risk for the detection of *Escherichia coli* and *Trueperella pyogenes* in grazing dairy cows (n = 55)

	Bacterial growth^a^		
	% (n)	OR^b^ (95% CI)	P
Vaginal discharge (VD)			0.06
VD0	39 (7/18)	Referent	
VD1	25 (3/12)	0.64 (0.21–2.01)	
VD2	60 (6/10)	1.54 (0.71–3.33)	
VD3	73 (11/15)	1.88 (0.98–3.63)	

^a^Determined by routine bacteriological testing

^b^OR (95% CI): Odds ratio and 95% confidence interval

## Discussion

FTIR spectroscopy has been described as a diagnostic technique that combines high specificity, low cost and easy usage. Most studies, however, compared this technique with complex and costly molecular techniques [19, 33–35], and not with common simple routine testing methods. Our study assessed FTIR spectroscopy as an alternative to the routine methods of bacteriological identification that are commonly used by laboratories, trying to propose a more efficient tool that provides results faster (approximately 1 day) and at lower costs [36]. Despite that, there were four cases in which the results by routine methods of bacteriological identification differed from the results by FTIR spectroscopy. We found that FTIR spectroscopy had a good agreement with routine bacteriological testing methods for identification of well-known uterine pathogens such as *E. coli* and *T. pyogenes*. This is in agreement with recent reports that showed that FTIR spectroscopy improves and facilitates the identification of bacteria in cases of mastitis in dairy cows compared with routine mastitis diagnostics [36]. We do not have an explanation for the observed differences in identification for the 2 *E coli* and 2 *T. pyogenes* samples. However, the reliability of the obtained results mainly were dependent on the reference database used in FTIR spectroscopy [37], and on the operators’ skills at both locations with routine methods of bacteriological identification [38]. Although FTIR spectroscopy is not an on-farm test, which would be preferred for field practice, time from sampling to diagnosis was approximately one day shorter than for routine methods of bacteriological identification. Furthermore, FTIR spectroscopy is a high throughput technique, i.e. it takes less work per sample [39]. Although FTIR spectroscopy has been used to identify uterine bacteria [7, 10], to the authors’ knowledge, this is the first study evaluating the use of FTIR spectroscopy together with traditional bacteriological identification methods performed on uterine samples from dairy cows. Further studies are required to confirm these findings with a larger number of samples.

It has been established that FTIR spectroscopy has sufficient resolution power to distinguish between different taxonomic ranks [40, 41]. Furthermore, the specificity of FTIR method is so high that it is able to identify the changes in bacteria’s biochemical composition associated with cultivation in different media [30], with mutational process or even with the variation related to the adaptation to the environment of host animal [23, 33, 42, 43]. Therefore, the success of the FTIR spectroscopy is dependent highly on the complexity of the reference spectrum library [33, 41]. In fact, we have been successful in detecting uterine bacteria in Argentinean dairy cows that are reared under a rotationally grazing system, by using one spectral database containing only spectra from uterine bacteria from Austrian and German dairy cows kept in free-stall facilities. A limitation of the present study is that it did not involve anaerobic uterine bacteria. A comparison between anaerobic uterine bacteria obtained from Austrian–German dairy cows and Argentinean dairy would have been interesting and informative. The main reason for that limitation was the difficulty to obtain reliable isolates under Argentinean extensive field conditions. Furthermore, it would be interesting for further research to study the changes in the FTIR spectra from bacteria before and after becoming resistant to antibiotic therapy. In that case, FTIR spectroscopy could become a useful tool to identify resistant uterine bacteria, to choose pathogen-specific antibiotics, or even to forego using antibiotics and choose an alternative treatment [44, 45].

In the present study, isolation of well-known uterine pathogens *E. coli* and *T. pyogenes* tended to increase with VD score. This finding is in agreement with previous studies [27] that found a correlation between isolation of recognized pathogens (*E. coli*, *T. pyogenes, F. necrophorum* and *P. melaninogenicus*) and VD score. Some researchers [27, 28], however, found significant correlation between *T. pyogenes* and VD score (P < 0.001), but not between *E. coli* and VD score (P = 0.21). Unexpectedly, a high number of isolates was observed in cows with VD0, although it was still lower in VD0 and VD1 compared with VD2 and VD3. This finding is in agreement with Wagener et al. [10] who detected *E. coli* and *T. pyogenes* in uterine samples from cows VD0 and VD1 less frequently than in cows with VD2 and VD3. Another finding was that no bacteria could be isolated from half of the tested cows. Among the possible explanations is that infection was not present or that the bacteria were not cultivable by the used method.

## Conclusions

The presence of *E. coli* and *T. pyogenes* in uterine samples from Argentinean dairy cows under an extensive pasture based management system can be detected by using FTIR with a database built with uterine bacteria from European dairy cows. Future studies are needed to determine if FTIR can be used as an alternative to routine bacteriological testing methods.
